# Dynamic right ventricular outflow tract obstruction caused by a large interventricular membranous septal aneurysm

**DOI:** 10.1007/s12471-018-1150-0

**Published:** 2018-08-23

**Authors:** L. Velicki, D. G. Jakovljevic, A. M. Milosavljevic, M. Todic, J. Rajic, M. Fabri

**Affiliations:** 1 Faculty of Medicine, University of Novi Sad, Novi Sad, Serbia; 2 Institute of Cardiovascular Diseases Vojvodina, Clinic for Cardiovascular Surgery, Sremska Kamenica, Serbia; 3 Faculty of Medical Sciences, Newcastle University and Newcastle upon Tyne Hospitals NHS Foundation Trust, Newcastle upon Tyne, UK

A 60-year-old female patient was evaluated for progressive dyspnoea. Twenty-six years ago, the patient underwent atrial septum defect closure and pulmonary valve commissurotomy.

Transthoracic echocardiography showed preserved left heart function with normal endocavitary dimensions and moderate aortic regurgitation (pressure half time 320 ms, vena contracta 0.6 cm, regurgitant volume 0.5 ml, effective regurgitant orifice area 0.25 cm^2^). Echocardiography demonstrated increased velocity (maximal 4.8 m/s, velocity time integral 122.1 cm) with pressure gradients (maximal 92 mm Hg, mean 38 mm Hg) over the right ventricular outflow tract (RVOT), which was narrowed to 0.8 cm. Transoesophageal echocardiography established the cause of the RVOT obstruction: a large (3 × 2 cm) interventricular membranous septal aneurysm (IMSA) causing dynamic infundibular obstruction (Fig. 1, and Video 1 and 2). The patient underwent aortic valve replacement and transaortic plication of the IMSA with two pledgeted sutures.

**Fig. 1:**
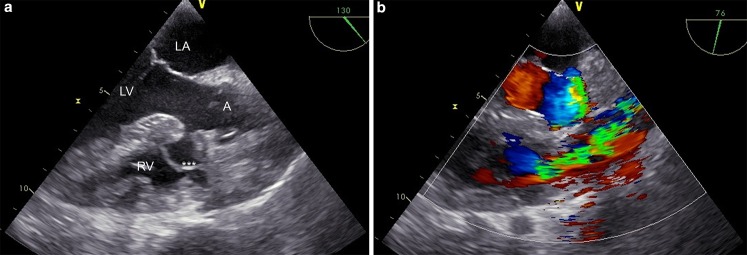
Transoesophageal echocardiography. **a** Membranous septum aneurysm dynamically obstructing RVOT during systole. See also online videos; **b** RVOT Doppler tracings showing signs of stenosis (maximal gradient 92 mm Hg, mean gradient 38 mm Hg). (*LA* left atrium, *LV* left ventricle, *A* aorta, *RV* right ventricle. ^***^Interventricular membranous septal aneurysm)

Dynamic systolic RVOT obstruction is one of the most unusual complications associated with IMSA with only a few cases reported so far [1–5].

## Caption Electronic Supplementary Material

Video 1 Transoesophageal echocardiography of RVOT at the same level as depicted in Fig. 1

Video 2 Transoesophageal echocardiography: RVOT Doppler tracings
